# Substance Use Disorder Is Associated With Alcohol-Associated Liver Disease in Patients With Alcohol Use Disorder

**DOI:** 10.1016/j.gastha.2022.02.004

**Published:** 2022-03-30

**Authors:** Augustin G.L. Vannier, Vladislav Fomin, Raymond T. Chung, Suraj J. Patel, Esperance Schaefer, Russell P. Goodman, Jay Luther

**Affiliations:** 1 MGH Alcohol Liver Center, Massachusetts General Hospital, Harvard Medical School, Boston, Massachusetts; 2 Gastrointestinal Unit, Department of Medicine, Massachusetts General Hospital, Harvard Medical School, Boston, Massachusetts; 3 Department of Medicine, Massachusetts General Hospital, Harvard Medical School, Boston, Massachusetts

**Keywords:** Alcohol abuse, Alcohol dependence, Substance abuse, Liver disease

## Abstract

**Background and Aims:**

Substance use disorder (SUD) commonly associates with alcohol use disorder (AUD), and certain substances have independently been shown to drive liver injury. In this work, we sought to determine if coexisting SUD in patients with AUD is associated with the presence of alcohol-associated liver disease (ALD).

**Methods:**

We performed a cross-sectional analysis using the Mass General Brigham Biobank to identify patients based on International Classification of Diseases, Tenth Revision, codes. We performed multivariate analyses accounting for a wide range of demographic and clinical variables to evaluate the association between SUD and ALD. We subsequently used the same method to evaluate the association between SUD and hepatic decompensation.

**Results:**

We identified 2848 patients with a diagnosis of AUD; 9.0% of them had ALD, and 25.2% had a history of SUD. In multivariate analyses, patients with SUD were more frequently diagnosed with ALD than those without SUD (odds ratio [OR] = 1.95, *P* = .001). Furthermore, the number of concurrent SUDs was positively associated with the diagnosis of ALD (OR = 1.33, *P* < .001). Independent of the presence of other SUDs, opioid use disorder in patients with AUD was associated with ALD (OR = 1.902, *P* = .02). In subsequent analyses, we found that sedative use disorder was associated with hepatic decompensation (OR = 2.068, *P* = .03).

**Conclusion:**

In patients with AUD, SUD, and particularly opioid use disorder, was independently associated with the diagnosis of ALD.

## Introduction

Excessive alcohol use is highly prevalent and contributes to significant morbidity and mortality in society.^1^, ^2^, ^3^ It is well-established that excessive alcohol intake increases the risk for developing alcohol-associated liver disease (ALD),^4^^,^^5^ a leading cause of morbidity among alcohol users.^5^ While most patients with excessive alcohol use do not experience severe liver injury, a minority can develop alcohol-related hepatitis or cirrhosis. Severe alcohol-related hepatitis, the most aggressive form of ALD, carries a dismal prognosis.^6^, ^7^, ^8^ Similarly, patients with cirrhosis, and in particular, those who have experienced a liver-related decompensating event, face high 2-year mortality rates.^9^ Accordingly, identifying modifiable risk factors for advanced liver disease among patients with alcohol use disorder (AUD) is critical.

In recent years, the use of nonalcohol substances, such as marijuana, opioids, benzodiazepines, and cocaine, has increased and resuled in significant burden on patients and the medical system.^10^^,^^11^ Due to common epidemiologic, socioeconomic, and psychiatric drivers, there is frequently a comorbidity between AUD and other substance use disorders (SUDs).^12^, ^13^, ^14^, ^15^ In addition, it has been demonstrated that hospitalized patients with ALD have a significantly higher prevalence of nonalcohol SUDs than hospitalized patients without underlying liver diseases.^16^ Notably, for each of these substances, there exist mechanistic data demonstrating their ability to independently drive hepatotoxicity, thereby potentially accentuating the negative effect of alcohol on the liver in patients with AUD.^17^, ^18^, ^19^, ^20^

Because of the increased frequency of nonalcohol substance use in patients with AUD and the potential hepatotoxicity of these substances, we hypothesize that patients with AUD and other substance use history will be more frequently diagnosed with ALD than those with AUD and no history of other substance use. In this work, we leverage a large and well-characterized cohort of patients with AUD who exhibit varying degrees of liver injury and substance use patterns to examine the association between substance use and the presence and severity of ALD.

## Methods

### Patient Cohort

For this cross-sectional associative study, we identified eligible patients in the Massachusetts General Brigham (MGB) Biobank,^21^ which is a large integrated database comprised of over 125,000 consented subjects within the MGB health-care system. Robust demographic, clinical, social, and demographic data are available for each patient and accessible through an online platform. Data are derived from the electronic health record and health surveys. All patients included in the database provided informed consent. To identify patients with AUD, we queried the MGB Biobank for patients with the following International Classification of Diseases, Tenth Revision, (ICD-10) diagnoses: alcohol abuse (F10.1) or alcohol dependence (F10.2). We included all patients with one of these diagnoses that also completed an alcohol questionnaire to quantify their alcohol use.

### Demographic and Clinical Factors

We collected the following demographic and clinical data on our cohort: sex, age, race, ethnicity, body mass index, viral hepatitis status, drinking history, history of nicotine dependence, substance use history, psychiatric disorder history, and the receipt of medical addiction therapy or psychotherapy (Table A1). For lab testing, the most recent values were used in our analyses. Alcohol consumption was quantified from a patient-completed questionnaire, which asked the following question: “During the past year, how many alcoholic drinks (glass/bottle/can of beer; 4oz glass of wine; drink or shot of liquor) did you usually drink in a typical week?”. Participants were given the following options: (1) none, or less than one per month, (2) 1–3 per month, (3) 2–4 per week, (4) 5–6 per week, (5) 1–2 per day, (6) 3–4 per day, (7) 5–6 per day, or (9) more than 6 per day. We converted these categorical responses into a linear score (0–8) for our multivariate analyses. Viral hepatitis was assessed through analysis of lab testing and diagnosis history. Patients were classified as having hepatitis B if they had a positive hepatitis B antigen test or a diagnosis of chronic hepatitis B in their medical chart. Patients were classified as having hepatitis C if they had a positive hepatitis C antibody test or a diagnosis of hepatitis C in their medical chart. Patients that were missing either a hepatitis C antibody test or a hepatitis B antigen test and were not diagnosed with viral hepatitis were considered untested for the purpose of our multivariate analyses.

### Definition of SUD

We identified the subset of patients with AUD who also had a history of a SUD. We determined that patients had a SUD if they had an ICD-10 code in their chart that supported a diagnosis of *abuse* of or *dependence* of the following substances: cocaine (F14.1 & F14.2), opioids (F11.1 & F11.2), cannabis (F12.1 & F12.2), other stimulant (F15.1 & F15.2), inhalants (F18.1 & F18.2), and sedatives, hypnotic or anxiolytic (F13.1 & F13.2). Stimulants include substances such as amphetamines, while sedatives, hypnotic or anxiolytic, refer to benzodiazepines and barbiturates.

### Definitions of ALD

We identified patients with ALD by looking for one of the following ICD-10 diagnoses: alcoholic hepatitis (K70.1), alcoholic fibrosis and sclerosis (K70.2), alcoholic cirrhosis of liver (K70.3), alcoholic hepatic failure (K70.4), other cirrhosis (K74.69), or unspecified cirrhosis (K74.60). We did not include patients with only an ICD diagnosis of either alcoholic fatty liver (K70.0) or alcoholic liver disease-unspecified (K70.9), as a high number of patients with moderate and extensive alcohol use develop hepatic steatosis, which is of unclear clinical significance with regard to liver-related morbidity and mortality.^22^ Hepatic decompensation was defined by a diagnosis of hepatorenal (K76.7) or hepatopulmonary syndrome (K76.81), hepatic encephalopathy/failure (K72.0, K72.1, K72.2, K72.9), hepatocellular carcinoma (C22.0), ascites (R18), and esophageal varices with bleeding (I85.01, I85.11).

### Statistical Analyses

Continuous variables were summarized using means compared using the t test with Welch correction; categorical variables were expressed as numbers and percentages and compared using the Fisher exact test. We also performed univariate logistic regressions to determine the odds ratio (OR) and 95% confidence interval of having ALD for each risk factor, demographic characteristic, and SUD. Multivariate logistic regressions were adjusted for the following variables: demographics, risk factors for AUD (homelessness, psychiatric disorders, nonalcohol SUDs), specific AUD diagnosis (abuse, dependence or both), the receipt of medical addiction therapy, psychotherapy, alcohol use history (as assessed by questionnaire), and concurrent liver diseases that may contribute to the development and progression of ALD (hepatitis B and hepatitis C virus positivity, nonalcoholic steatohepatitis, biliary cholangitis, autoimmune hepatitis, chronic passive liver congestion, alpha-1 antitrypsin deficiency, and hemochromatosis). All analyses were conducted using GraphPad Prism (San Diego, CA).

## Results

### Patient Demographics

Our analysis consisted of 2848 patients with AUD who had completed an alcohol use questionnaire (Figure). Demographic data are provided in Table 1. We found no difference in the severity of AUD, based on diagnostic coding of alcohol abuse, in patients with AUD with or without SUD (81% vs 78%, respectively, *P* > .05). Many patients had multiple SUDs (Figure A1)

**Figure:**
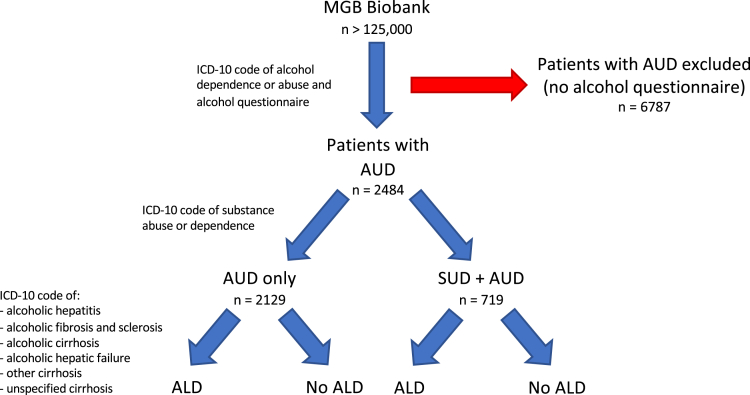
Flowchart of patient selection. The flow diagram details the method for patient selection in our analyses.

**Table 1:** Demographics of the Cohort

Variable	All patients (2848)	AUD (2129)	AUD + SUD (719)	*P* value^a^
Age	56.7	59.0	49.9	<.001
Male sex (%)	1574 (55.2%)	1185 (55.6%)	389 (54.1%)	.5
White (%)	2524 (88.6%)	1909 (89.6%)	615 (85.5%)	.003
BMI	28.5	28.5	28.3	.5
Viral hepatitis	209 (7.3%)	100 (4.6%)	109 (15.1%)	<.001
Homeless (%)	112 (3.9%)	28 (1.3%)	84 (11.6%)	<.001
Psychotherapy (%)	1102 (38.6%)	649 (30.4%)	453 (63%)	<.001
Psychiatric disorder (%)	2374 (83.3%)	1686 (79.1%)	688 (95.6%)	<.001
Nicotine dependence (%)	994 (34.9%)	609 (28.6%)	385 (53.5%)	<.001
MAT (%)	1113 (39%)	724 (34%)	389 (54.1%)	<.001

BMI, body mass index; MAT, medical addiction therapy.

a All *P* values refer to a Fisher exact test (categorical variables) or a t test with Welch correction (linear variables) comparing patients with both SUD and AUD to those with only AUD.

### SUD Is Associated With the Presence of ALD in Patients With AUD

Given the potential biological contribution of substance use to liver disease,^17^, ^18^, ^19^, ^20^ we first sought to determine if there was an association between the presence of SUD in patients with AUD and a diagnosis of ALD. We found that patients with a history of any drug disorder were more often diagnosed with ALD than those without a history of a drug disorder (OR = 1.95, *P* = .001) (Table 2). In subgroup analyses, AUD patients with concurrent opioid disorder (OR = 1.90, *P* = .01) were more frequently diagnosed with ALD. Notably, we found no association between ALD and sedative, stimulant, cocaine, or cannabis disorder (Table 2). Furthermore, we found a positive association between the number of SUDs and the diagnosis of ALD (OR = 1.33, *P* < .001). Taken together, we found a positive association between the presence of SUD and ALD in patients with AUD, especially in those patients with a history of opioid disorder.

**Table 2:** SUD Is Independently Associated With Increased Odds of Having Alcohol-Associated Liver Disease in Patients With AUD

Substance use disorder	Odds ratio	95% CI	*P* value
Any SUD	1.95	1.31–2.89	.001
Number of SUDs	1.33	1.14–1.55	<.001
Cannabis	1.57	0.92–2.63	.09
Cocaine	1.33	0.72–2.40	.4
Inhalant	1.21	0.66–2.18	.5
Opioid	1.90	1.13–3.17	.01
Other	0.50	0.18–1.26	.2
Sedative	1.25	0.59–2.56	.6

CI, confidence interval.

### Sedative Use Disorder, But Not Other Substance Use Diagnoses, Associates With Hepatic Decompensation in Patients With AUD

Given the wide spectrum of ALD, we next sought to determine if there was an association between SUD and hepatic decompensation in patients with AUD. While we did not find an association between SUD and hepatic decompensation (OR = 1.08, *P* = .68), there was a positive association between sedative use disorder and hepatic decompensation (OR = 2.07, *P* = .03) (Table 3). We did not find an association between ALD and the number of SUDs or the presence of cannabis, cocaine, inhalant, or sedative use disorder. Taken together, we conclude that a diagnosis of sedative use disorder was associated with hepatic decompensation in patients with AUD.

**Table 3:** Sedative Use Disorder, But Not Other SUDs, Is Independently Associated With Increased Odds of Hepatic Decompensation in Patients With AUD

Substance use disorder	Odds ratio	95% CI	*P* value
Any SUD	1.08	0.74–1.56	.7
Number of SUDs	1.11	0.94–1.29	.2
Cannabis	0.91	0.53–1.50	.7
Cocaine	0.84	0.45–1.50	.6
Inhalant	1.23	0.68–2.16	.5
Opioid	1.05	0.62–1.74	.9
Other	1.08	0.43–2.43	.9
Sedative	2.07	1.04–3.95	.03

CI, confidence interval.

## Discussion

In this cross-sectional study, we found a positive association between the presence of ALD and a history of SUD in a large cohort of patients with AUD. Notably, opioid use disorder significantly associated with the presence of ALD. Moreover, we found that an increasing number of SUD diagnoses in a patient with AUD also associated with ALD. Finally, we also found that sedative use disorder, but not other SUDs, associated with hepatic decompensation in patients with AUD. These associations were identified in multivariate analyses, suggesting drug use may independently contribute to liver injury in patients with AUD.

It is well established that there is an association between alcohol use and liver disease, ranging from reversible hepatic steatosis to steatohepatitis and to chronic hepatic fibrosis. The wide spectrum of liver diseases observed in patients with AUD highlights the numerous factors independent of quantity of alcohol consumed that may contribute to liver injury. While many of these factors are nonmodifiable, there is an urgent and unmet need to identify modifiable risk factors in these patients. We hypothesized that concurrent drug use would represent a key variable in driving liver disease in patients with AUD. This hypothesis was based on established data that highlight the ability of certain substances to mechanistically drive liver injury. In particular, opioids are metabolized in the liver and may accentuate liver injury through lipid peroxidation and mitochondrial injury.^23^ Furthermore, hepatocytes have numerous opioid receptors that drive pathways that may contribute to liver injury.^24^ Opioids have been implicated in altering bile acid metabolism as well, which could potentially effect liver homeostasis.^25^ Accordingly, there is biological plausibility to support our observed findings that substance use associates with liver disease in patients with AUD.

Previous work has highlighted an increase in SUD prevalence among patients with ALD. An analysis of the National Inpatient Sample data from 2011 found an increased likelihood for hospitalized patients with ALD to have SUD compared with hospitalized patients without ALD.^16^ A more recent analysis of the National Inpatient Sample data found that compared with the patients with chronic liver disease, patients with comorbid chronic liver disease and SUD were more likely to have ALD.^11^ Our study builds on these data in multiple ways. First, we study the effect of SUD on liver diseases in only those patients with AUD. Second, we demonstrate the association of individual SUDs with ALD. Finally, we show the effect of SUDs on hepatic decompensation in patients with AUD.

Patients with AUD often use other recreational drugs. A recent cross-sectional study demonstrated that 8% of those who use alcohol engage in other drugs, and among those who have AUD, 15% have another SUD including hard drugs and marijuana.^26^ Those with concurrent SUDs tend to be male, have lower annual incomes, have more severe AUD, and tend to have more comorbid psychiatric disorders.^26^ In our analysis, most patients with AUD did not have a concurrent SUD, and controlling for alcohol use did not eliminate the association between hard drug use and liver disease.

It is impossible to examine the effect of drug use simply from a biologic perspective without recognizing the societal context that these patients live in and the stigma they experience. Many people who inject drugs feel judged by the health-care system and, even prior to engaging in it, feel like their complaints will be dismissed or they will be turned away.^27^^,^^28^ Studies utilizing qualitative interviews of people with SUD revealed that they use the coping strategies of delaying care or seeking care with alternative medicine.^28^ These feelings are not simply imagined; in reality, many health-care providers do harbor negative feelings toward people who use drugs.^29^ This results in reduced collaboration between professionals and patients, short and more task-oriented visits. Patients under these circumstances are less likely to complete treatment. Thus, even if drug use does not biologically contribute to liver disease, its stigmatizing effects can create a schism in health-care equity.^28^

Our study has several limitations. First, the observational and cross-sectional nature of our study limits the findings to associations and may have potential cofounders not accounted for on statistical analysis. This is an inherent limitation of an associative study; by adjusting for many factors in our multivariate analyses, we limit this effect as much as possible. Necessarily, however, we cannot be sure that we have completely avoided residual confounding. Second, the determination of AUD and SUD was made by ICD-10 codes, and each patient may not have been fully evaluated by a provider concentrating in addiction medicine. However, while detailed SUD histories taken by addiction specialists would have reduced the noise in our cohort, this should not sum to produce the associations that we demonstrated in this study. Rather, greater noise introduced by imperfect coding increases statistical uncertainty and makes such associations more difficult to demonstrate.

Despite these limitations, we conclude that in our cohort of patients with AUD, SUD was independently associated with the presence of ALD. Prospective cohort studies are warranted to conclusively establish the altered risk of patients with concurrent SUD and AUD to develop ALD.

## Supplementary Materials

Figure A1

Table A1
