# Supplementary figures and images for: Substance Use Disorder Is Associated With Alcohol-Associated Liver Disease in Patients With Alcohol Use Disorder
Source: Gastro Hep Adv. 2022 Mar 30;1(3):403–8. doi: 10.1016/j.gastha.2022.02.004 (PMC9038113; doi:10.1016/j.gastha.2022.02.004)

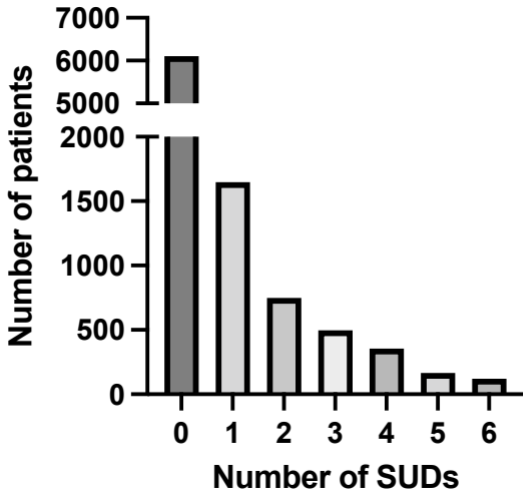

Supplement: Figure A1 [file mmc1.pdf]
