# Supplementary material for: Substance Use Disorder Is Associated With Alcohol-Associated Liver Disease in Patients With Alcohol Use Disorder
Source: Gastro Hep Adv. 2022 Mar 30;1(3):403–8. doi: 10.1016/j.gastha.2022.02.004 (PMC9038113; doi:10.1016/j.gastha.2022.02.004)
Supplement: Table A1 [file mmc2.docx]

Supplementary Table 1: Definitions of factors adjusted for in multivariate analyses

| Variable | Definition |
| --- | --- |
| Age | Continuous |
| Sex | M/F |
| Race | White/Black/Asian |
| Hispanic Ethnicity | Binary |
| Weight Class | Non-overweight, overweight, obese class I-III |
| Nicotine Dependence | ICD-10 code (F17.200) |
| Hepatitis B | ICD code (B18.0, B18.1) or a positive HBV antigen test or nonzero DNA test |
| Hepatitis C | ICD-10 code (B17.1, B18.2, B19.20) or a positive HCV antibody test, nonzero RNA test, or genotype test with a valid result) |
| Homelessness | ICD-10 code (Z59.0) |
| Psychotherapy | CPT4 code (90804-90815, 90826-90829, 90845, 90847, 90849, 90853, 90857, 90875, 90876, 99408) |
| Medical Addiction Therapy | 3 prescriptions of naltrexone, disulfiram, acamprosate, gabapentin, topiramate or baclofen before ALD |
| Alcohol Use History | Linear score based on number of drinks consumed per week |
| Cannabis Use Disorder | ICD-10 code (F12.1, F12.2) |
| Cocaine Use Disorder | ICD-10 code (F14.1, F14.2) |
| Inhalant Use Disorder | ICD-10 code (F18.1, F18.2) |
| Opioid Use Disorder | ICD-10 code (F11.1, F11.2) |
| Other Stimulant Use Disorder | ICD-10 code (F15.1, F15.2) |
| Sedative Use Disorder | ICD-10 code (F13.1, F13.2) |
| Anxiety Disorder | ICD-10 code (F40-F48) |
| Specific Personality Disorder | ICD-10 code (F60) |
| Mood Disorder | ICD-10 code (F30-F39) |
| Schizophrenia Disorder | ICD-10 code (F20-F29) |
| Chronic Passive Congestion of Liver | ICD-10 code (K76.1) |
| Hemochromatosis | ICD-10 code (E83.11) |
| Autoimmune Hepatitis | ICD-10 code (K75.4) |
| Primary Biliary Cirrhosis | ICD-10 code (K74.3) |
| Secondary Biliary Cirrhosis | ICD-10 code (K74.4) |
| Biliary Cirrhosis | ICD-10 code (K74.5) |
| Alpha-1 antitrypsin deficiency | ICD-10 code (E88.01) |
| Nonalcoholic Steatohepatitis | ICD-10 code (K75.81) |
